# Supplementary material for: Identification of triacylglycerol remodeling mechanism to synthesize unusual fatty acid containing oils
Source: Nat Commun. 2024 Apr 26;15:3547. doi: 10.1038/s41467-024-47995-x (PMC11053099; doi:10.1038/s41467-024-47995-x)
Supplement: Supplementary file 1 — Supplementary information [file 41467_2024_47995_MOESM1_ESM.pdf]

Supplementary information

Identification of triacylglycerol remodeling mechanism to synthesize unusual fatty acid containing oils

Prasad Parchuri[1†], Sajina Bhandari[1†], Abdul Azeez[1], Grace Chen[2], Kumiko Johnson[2], Jay Shockey[3], Andrei Smertenko[1], Philip D. Bates[1*]

[1]Institute of Biological Chemistry, Washington State University, Pullman, WA, 99164, USA
[2]United States Department of Agriculture, Agricultural Research Service, Western Regional Research Center, Albany, CA 94710, USA
[3]United States Department of Agriculture, Agricultural Research Service, Southern Regional Research Center, New Orleans, LA, USA 70124

[†]These authors contributed equally.

*Corresponding Author:
Philip D. Bates
100 Dairy Road, PO Box 647411
Pullman, WA 99164-7411
phil_bates@wsu.edu

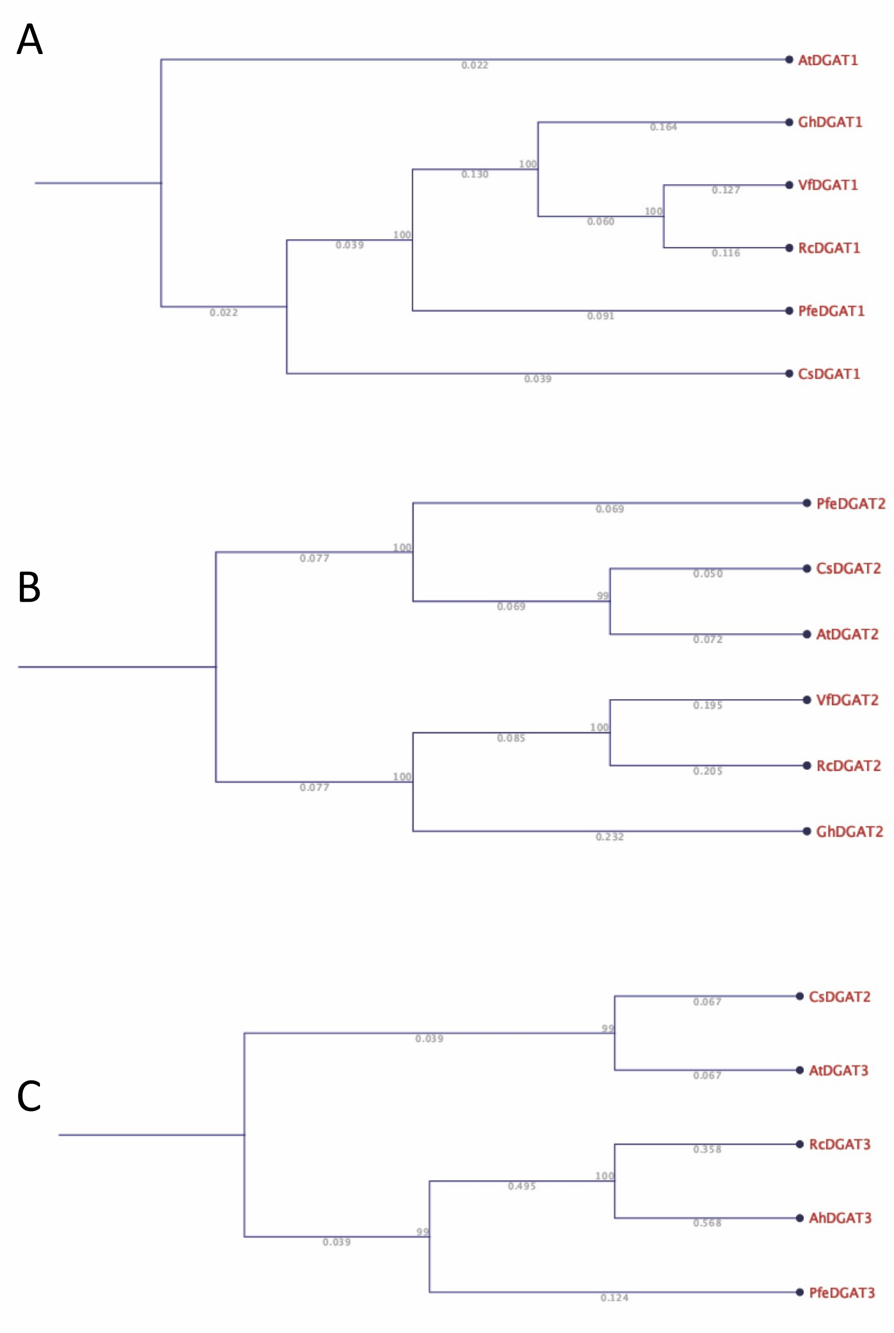

Supplemental Figure S1. Phylogenetic analysis of various DGAT1(A), DGAT2(B) and DGAT3(C) protein sequences from different plant species. *Pfe, Physaria fendleri; At, Arabidopsis thaliana; Vf, Vernicia fordii; Gh, Gossypium hirsutum; Rc, Ricinus communis; Cs, Camelina sativa; Ah, Arachis hypogea*. Branch length and bootstrap percentages are shown at the branches and nodes, respectively.

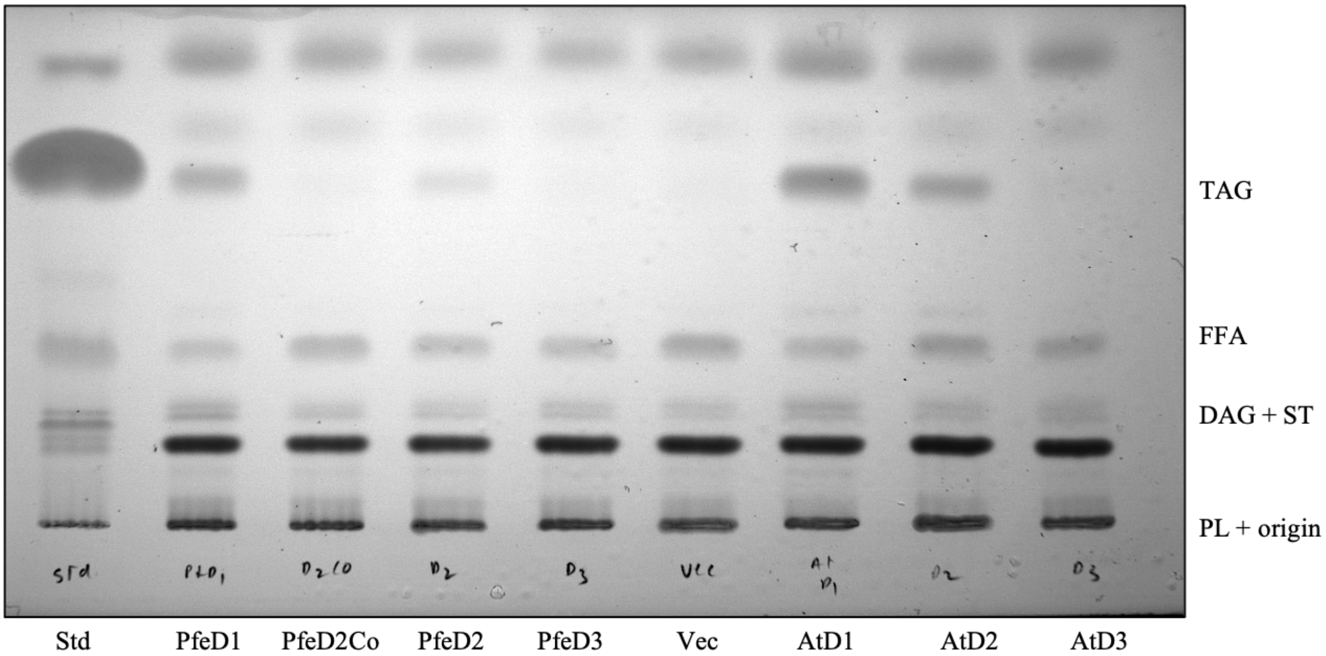

Supplemental Figure S2. Functional complementation of TAG biosynthesis in yeast by heterologous DGATs. The TLC represents the separation of neutral lipids of TAG deficient yeast mutant H1246 expressing *P. fendleri* and *A. thaliana* DGAT proteins. H1246 yeast cells expressing empty vector pYES2/NTC was used as control. Commercial seed oil was used as standard. TAG, triacylglycerol; FFA, free fatty acids; DAG, diacylglycerol; ST, sterol; and PL, phospholipids. PfeD2Co is a codon optimized version of PfeD2. The experiment was repeated thrice with similar results.

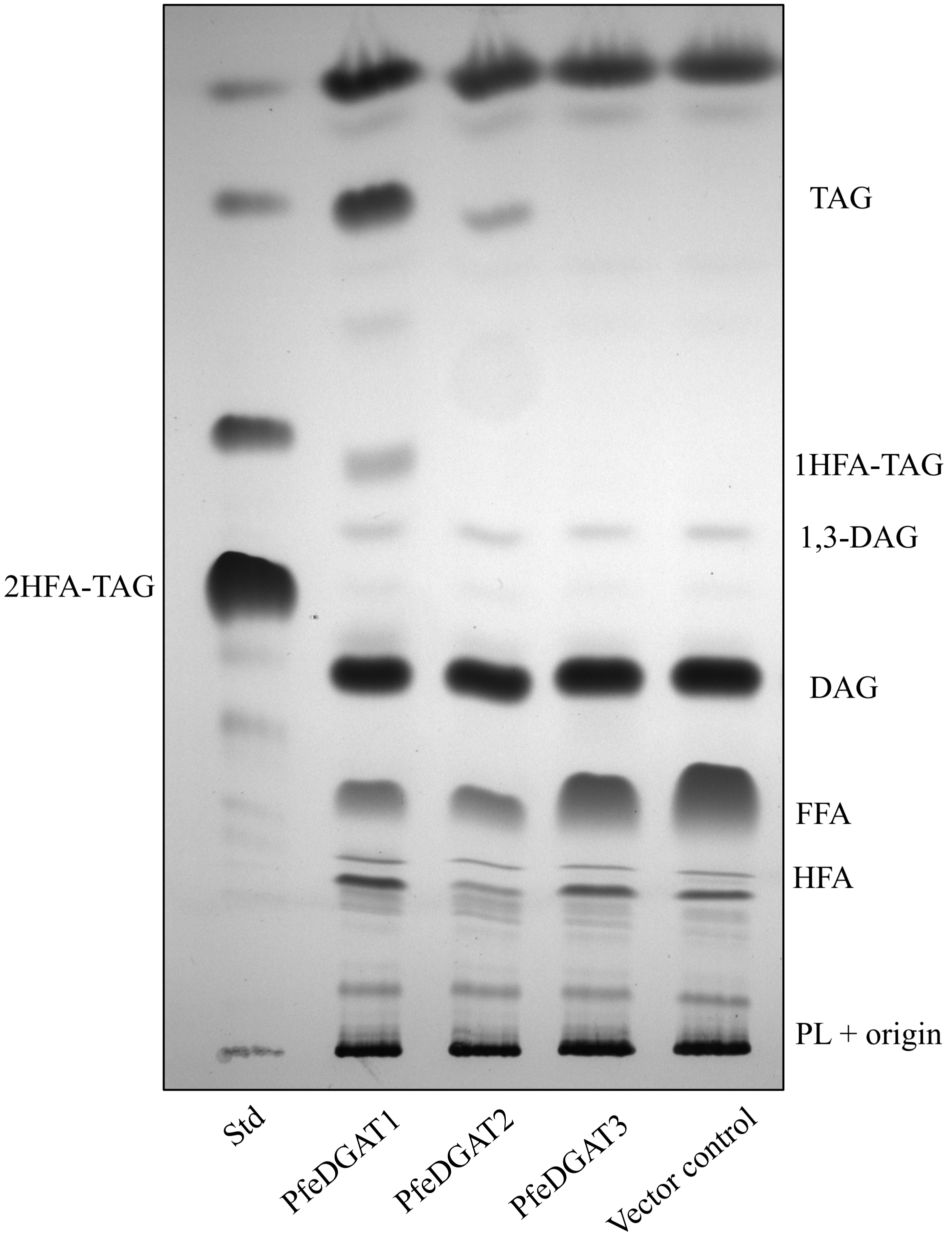

Supplemental Figure S3. Utilization of *P. fenderli* fatty acids for TAG synthesis in vivo by yeast mutant H1246 expressing *P. fenderli* DGAT enzymes. TLC represents the neutral lipid profile of transgenic yeast grown in the presence of 0.1% of Physaria seed oil (v/v), 0.5% of NP-40 and *Rhizopus mehei* lipase (1mg/ml). Yeast cells expressing empty vector pYES2/NTC was used as control. Physaria seed oil was used as standard. TAG, triacylglycerol; HFA, hydroxy fatty acid, FFA, free fatty acids; DAG, diacylglycerol; ST, sterol; and PL, phospholipids. The experiment was repeated twice with similar result s.

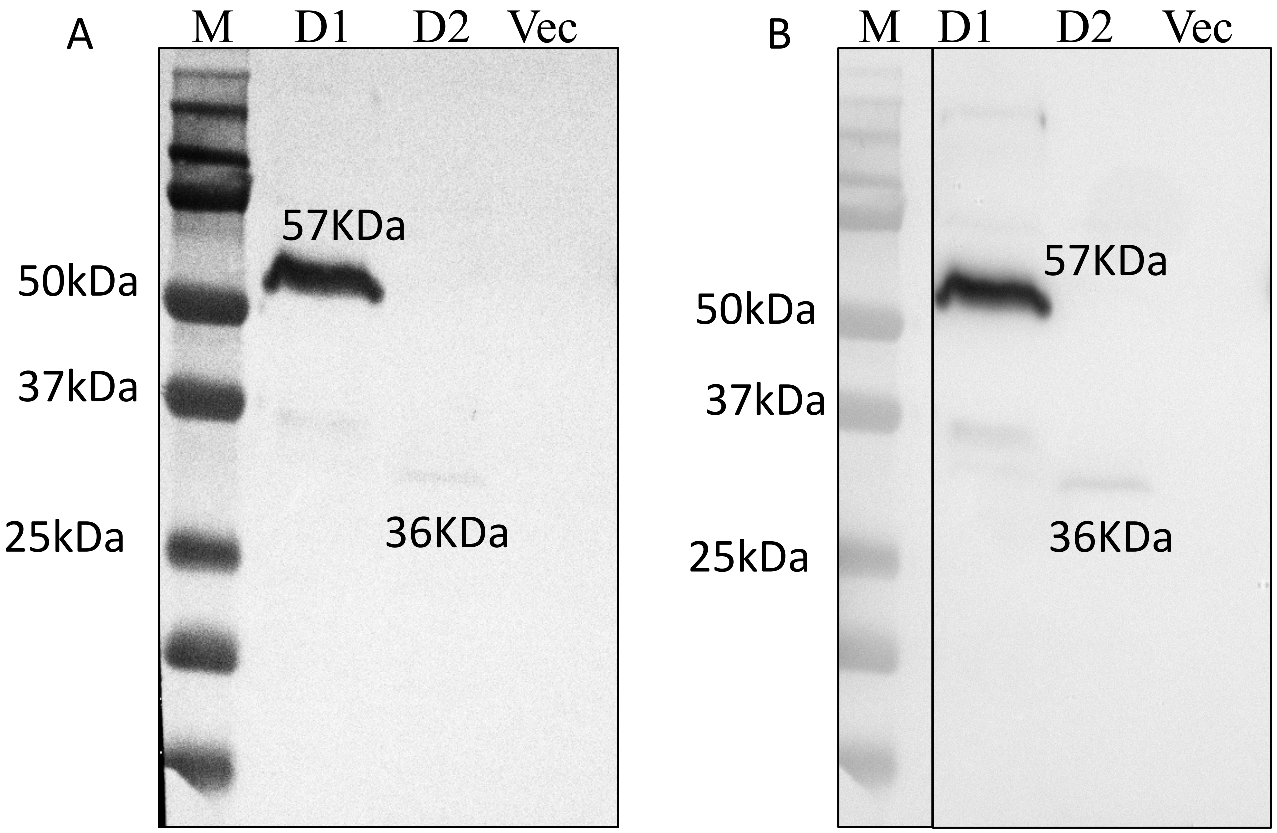

Supplemental Figure S4. Western blots demonstrating expression of His tagged PfeDGAT1 and PfeDGAT2 proteins in yeast microsomes. The blot was probed with anti-$(His)_6$ and HRP antibodies and was photographed without (A) and with (B) chemiluminescence exposure in the Gel Doc. The experiment was repeated twice with similar results.

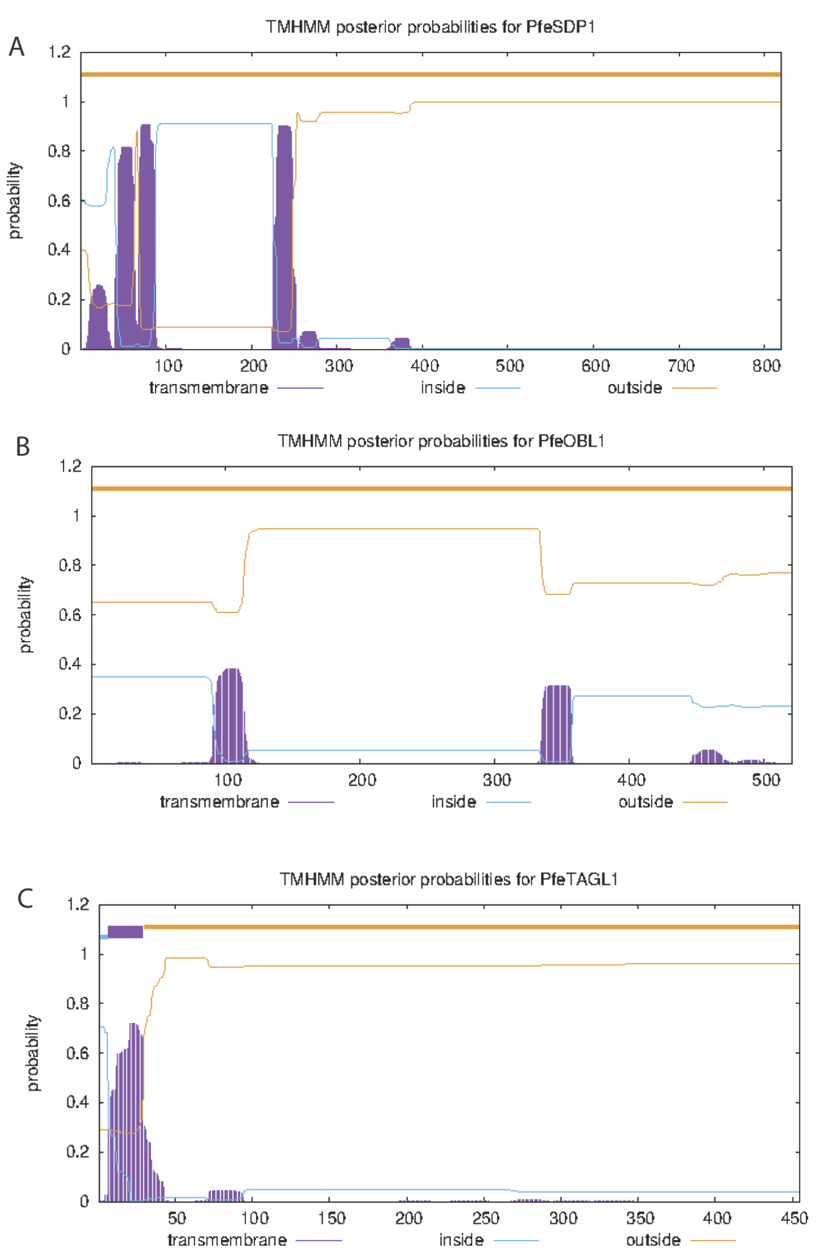

Supplemental figure S5. Hydrophobicity plots of SDP1, OBL1 and TAGL1 showing possibility of transmembrane domains. (A) PfeSDP1, (B) PfeOBL1, (C) PfeTAGL1.

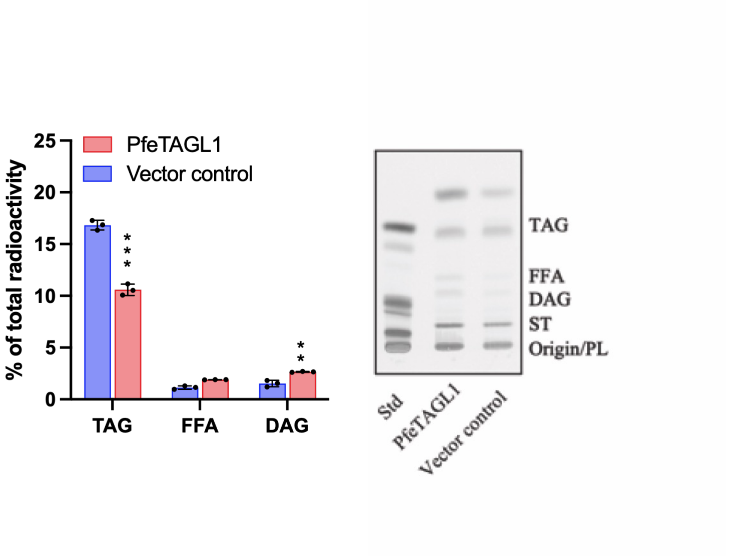

Supplemental Figure S6. Over expression of PfeTAGL1 alters neutral lipids in S. cerevisiae. The cells of *S. cerevisiae* over expressing PfeTAGL1 was induced with galactose in presence of [^{14}C]acetate (0.25 µCi/mL). A volume of induced cells equal to A_{600} = 50 was harvested and lipid were extracted. The relative amount of radioactivity in TAG, FFA, and DAG neutral lipids quantified by thin-layer chromatography (TLC) separation and phosphor imaging (insert). The results were expressed as mean ± SD of the three independent experiments and significance difference was determined using one-way ANOVA (***$p \leq 0.001$ and **$p \leq 0.002$).

A. PfeDGAT1-1 (RNAi-fragment, 206nt)

TACTACCGCGGCGGCCGCCTTCCTTTACCGTTGAGAAATTGGTACTTCAGAAGCTCATAT
CTGAGCCTGTTGTCATCATTCTTCATATTATTATCACCACGACAGCGGTTTTGTATCCAGT
TTACGTCACCCTAAGGTGTGATTCTGCCTTCTTATCAGGTGTCACATTGATGCTCCTCACT
TGCATTGTGTGGCTAAAGTTGGTTTCTTATGCTCACACGAACCCCGGGCTGCAGAAGTC

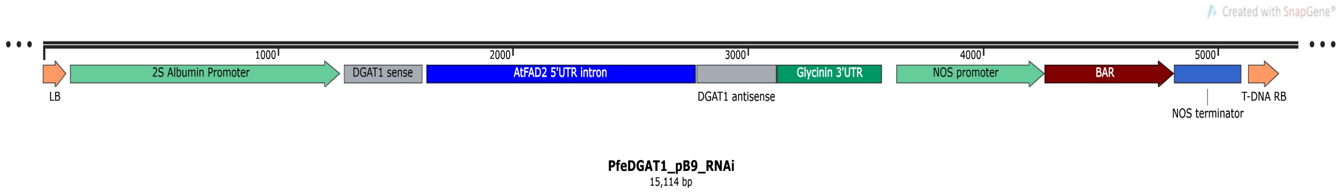

B. PfeDGAT2 (RNAi-fragment, 206nt)

TACTACCGCGGCGGCCGCTGGTGTCCAGGAGACTTATCATATGAAACATGACGTTGAGAC
CGTCTTTCTTTCCTCGAGAAGAGGATTTGTGCGCATAGCCATGGAACATGGGTGTCCTATA
GTTCCAGTTTTCTGCTTTGGGCAGTCACGGGTGTACAGTTGGTGGAAGCCAGATTGGGAT
CTCTATCTTAAATTATCAAGAGCAATCAGGTTTACCCCAATCTCCCGGGCTGCAGAAGTC

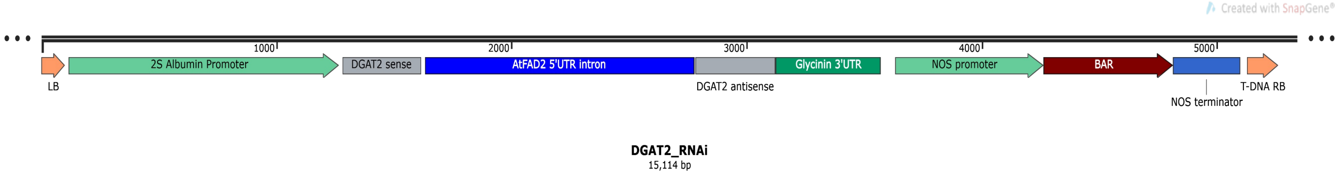

C. PfeTAGLipase like 1 (RNAi-fragment, 183nt)

TACTACCGCGGCGGCCGCACCTCTCGGGATGGGAATACATCCTCTGCTTTTCATCCGCG
TGTTGCAAATGAGTCATTGGCGATTTCCTCGGGATCTTCCTCTGCCTTATAAAGGATACA
GAGATGAAGATTGGCATGACAATGATGGAGCAGTGAACACTATATCAATGACTCACCC
GCGAATTCCCATCGAACATTCGAGCCCGGGCTGCAGAAGTC

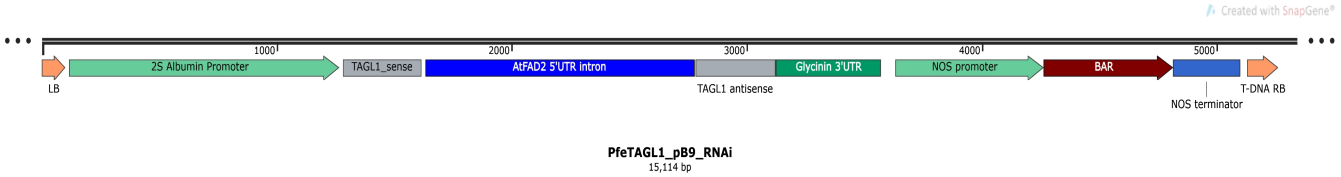

Supplemental Figure S7. The sequence of RNAi fragments and diagrams of RNAi constructs used in this study. (A) PfeDGAT1_RNAi fragment and construct, (B) PfeDGAT2_RNAi fragment and construct, (C) PfeTAGL1_RNAi fragment and construct. See methods section for construct preparation.

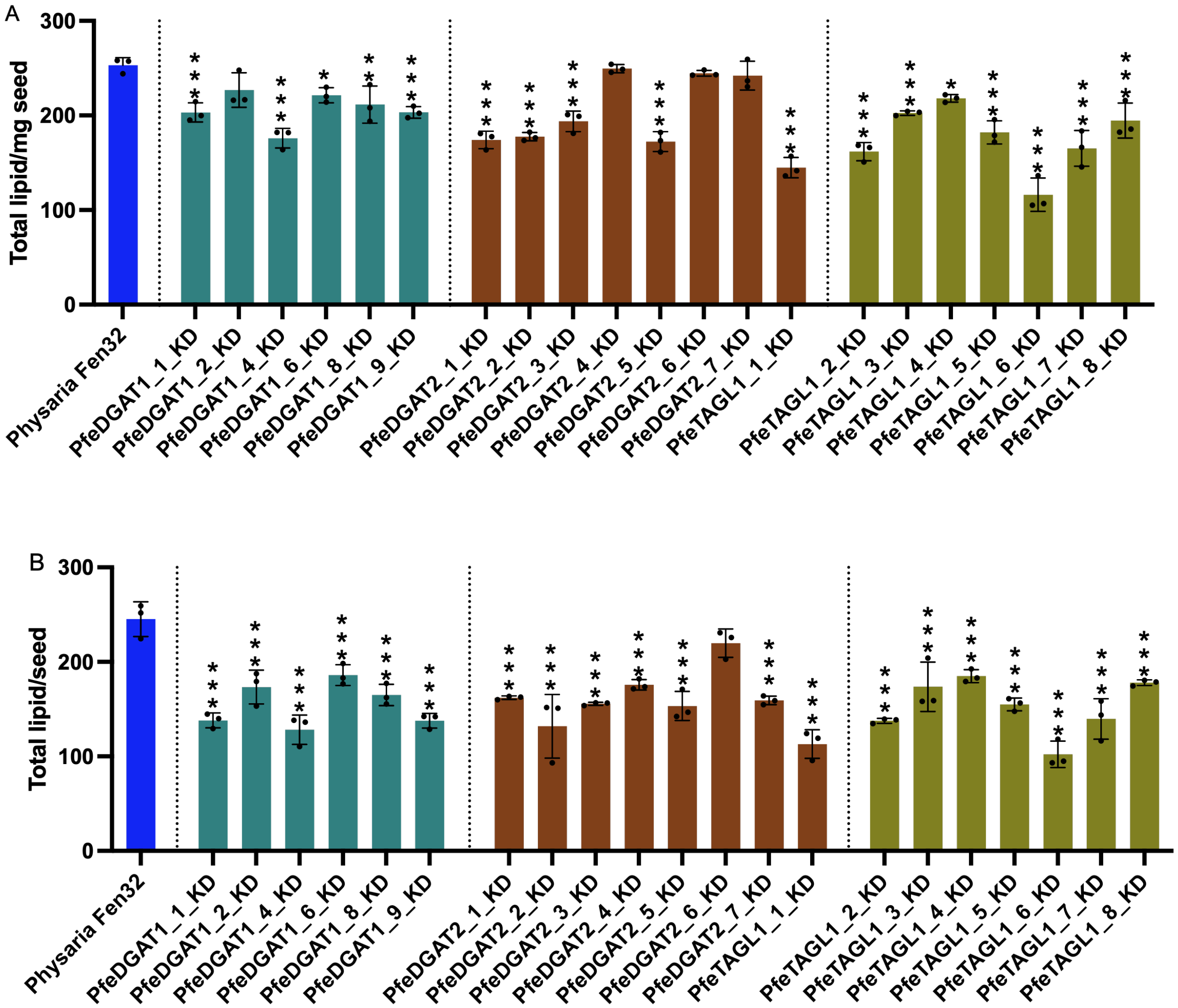

Supplementary Figure S8: Total lipid content of T2 seeds of PfeDGAT1, PfeDGAT2 and PfeTAGL1 knockdown lines. (A) Total lipid content on per mg seed basis. (B) Total lipid content on per seed basis. Physaria_fen32 was used as wildtype control. Data represents mean±SD (n= 3) of individual biological replicates. Lines significantly different ($p<0.05$) than Physraia_fen32 determined by one-way ANOVA with Dunnett's multiple comparisons correction are marked with a black asterisk. Source data underlying Supplementary fig.S8 A-B and exact p-values are provided in the source data file.

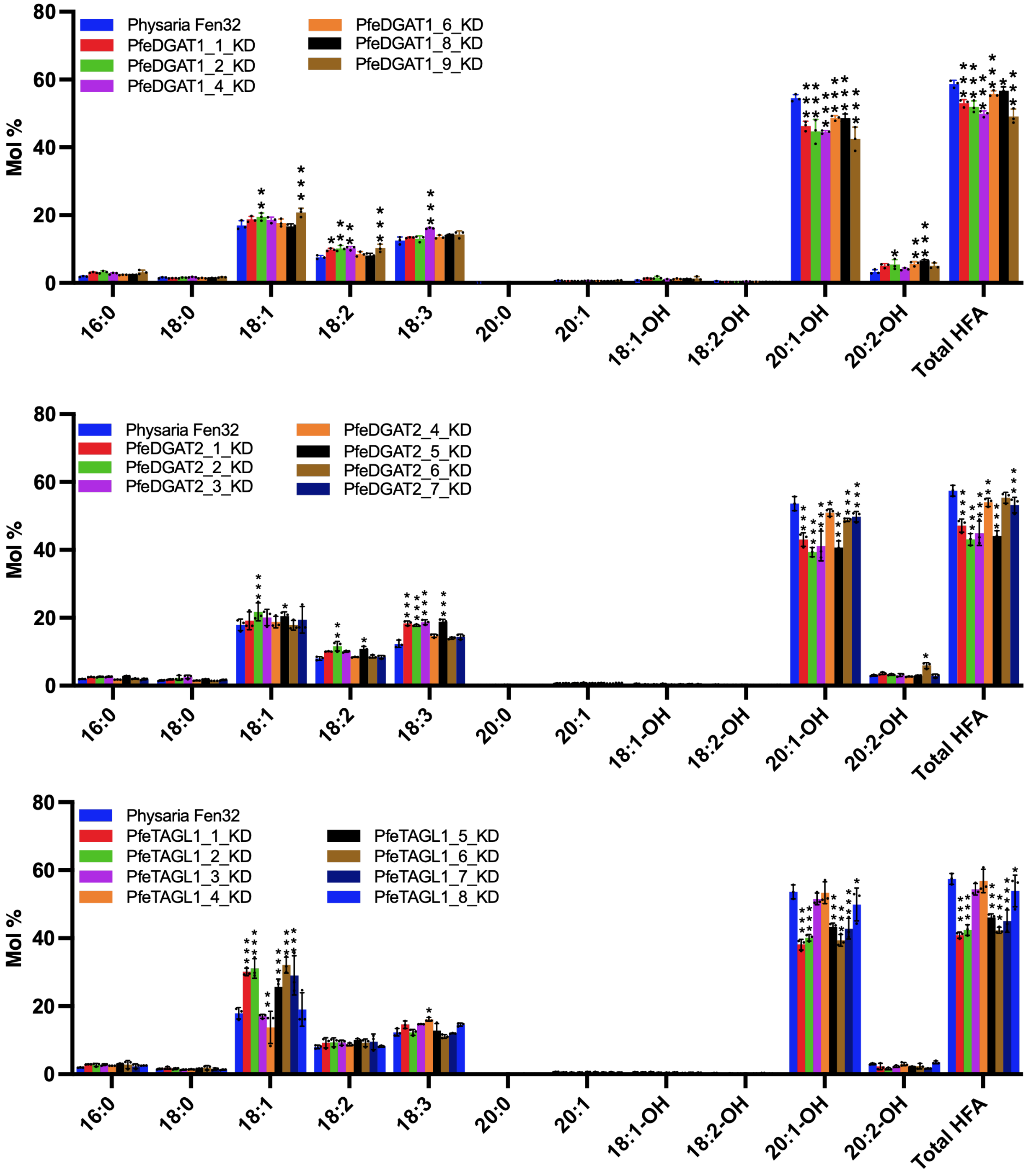

Supplementary Figure S9: Fatty acid composition of T2 seeds of PfeDGAT1, PfeDGAT2 and PfeTAGL1 knockdown lines. Physaria_fen32 was used as wildtype control. Data represents mean±SD (n= 3) of individual biological replicates. Lines significantly different (p<0.05) than Physraia_fen32 determined by two-way ANOVA with Dunnett's multiple comparisons correction are marked with a black asterisk. Source data underlying Supplementary fig.S9 A-B and exact p-values are provided in the source data file.

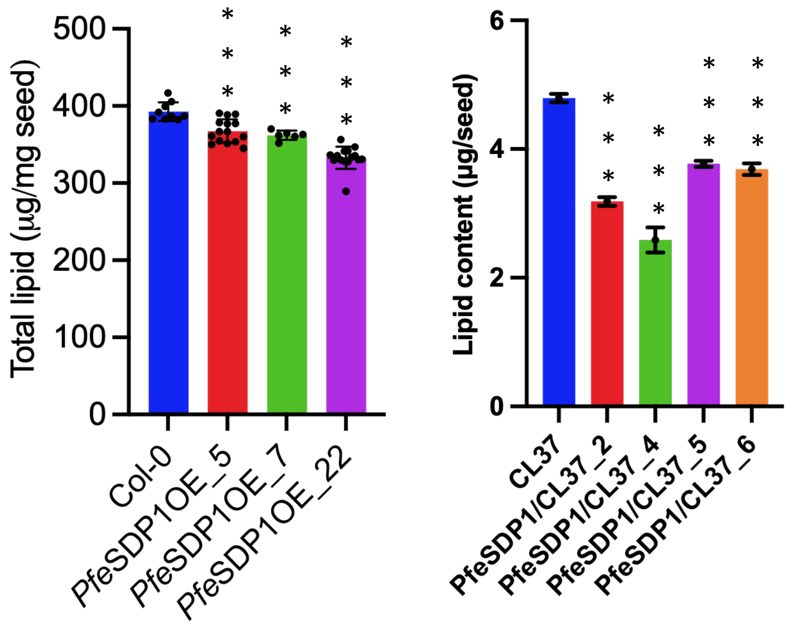

Supplemental Figure S10. Over expression of PfeSDP1 in Col0 and HFA producing Arabidopsis seeds (RcFAH/fae1, CL37). Left: Total lipid content of PfeSDP1 overexpression T4 lines in Col0 background , data represents at least 15 plants mean ± SEM per line. Right: Total lipid content of PfeSDP1 overexpression T2 lines in CL37 background, data represents three technical replicates mean ± SEM per line. Asterisks (**) and (***) indicate significant differences at P≤0.001 and P≤0.0001 compared to their respective control by two-tailed paired *t*-tests.

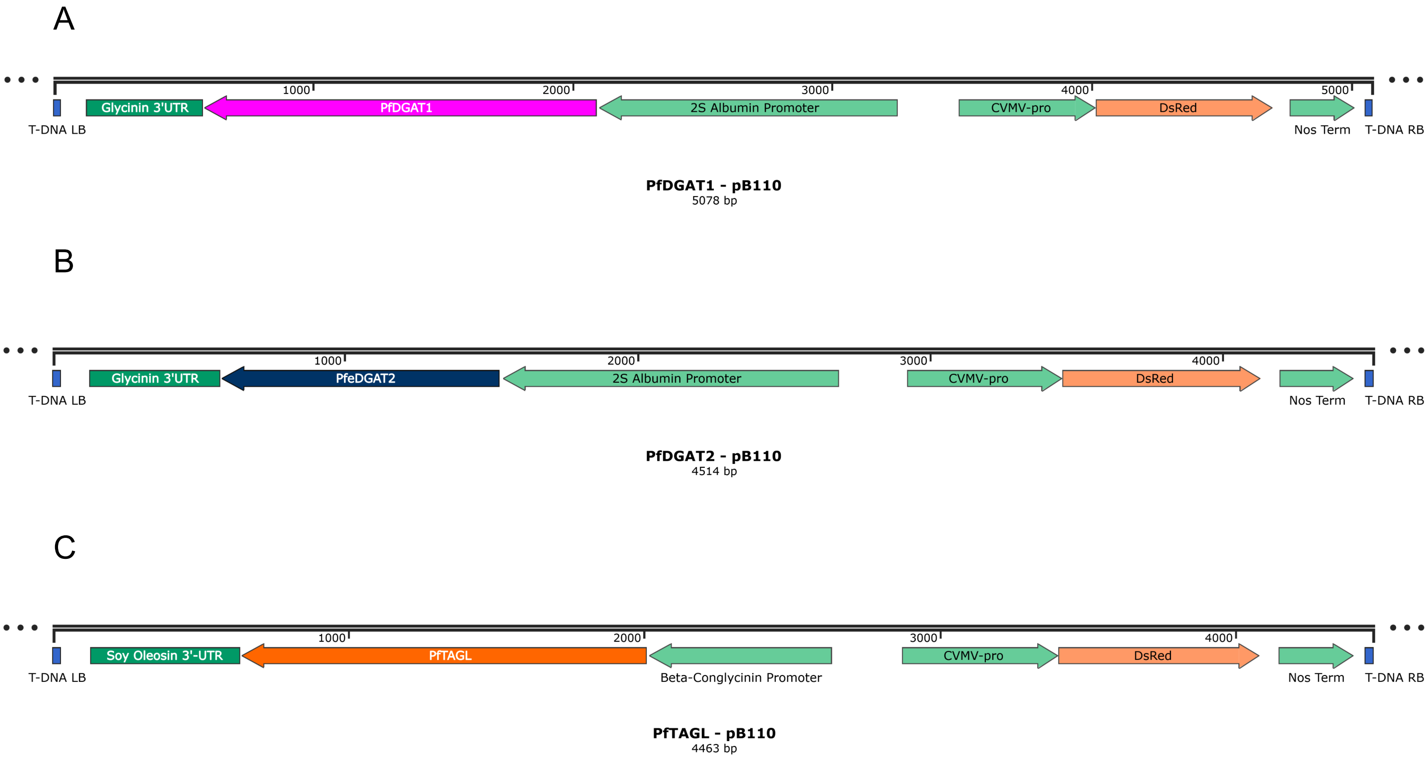

Supplemental Figure S11. Diagrams of plant transformation plasmids used in this study. The open reading frames for (A) PfDGAT1, (B) PfDGAT2, and (C) PfTAGL1 were cloned into the NotI and SacII sites of cloning vector pB34 (Shockey et al. 2015) which contains the Arabidopsis 2S-3 albumin promoter for strong, seed-specific expression in plants, and the soybean glycinin terminator. Promoter:gene:terminator cassettes were excised from the cloning vectors using AscI and cloned into the corresponding site in DsRed fluorescence-selectable plant binary vector pB110. For clarity, only the features contained within the T-DNA boundaries are shown, with the T-DNA left border on the left, and the right border on the right.

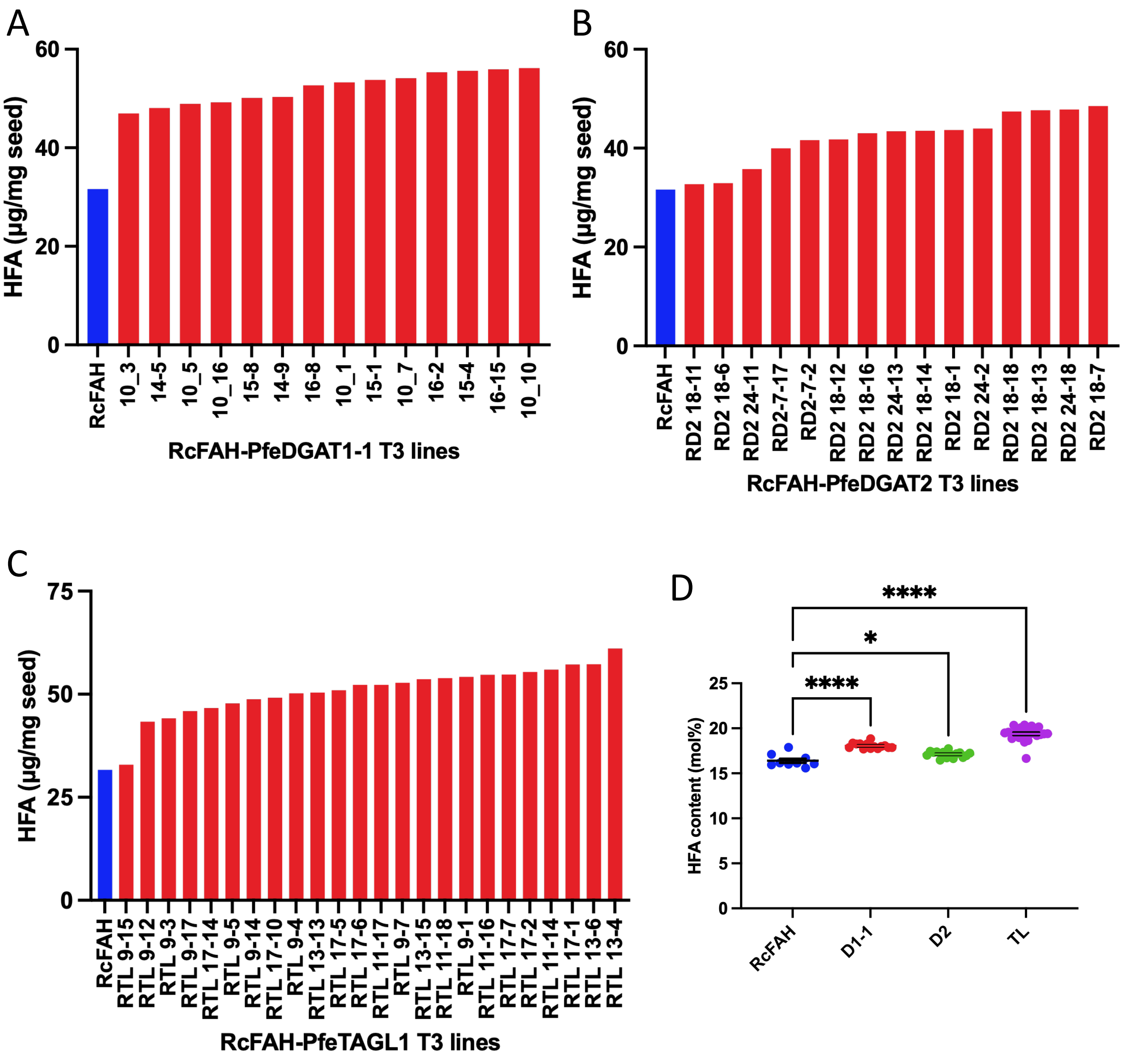

Supplemental Figure S12. HFA content (µg/mg seed) of T3 seeds of individual *Arabidopsis thaliana* RcFAH lines expressing *P. fendleri* TAG remodeling genes. (A) PfeDGAT1 (D1-1), (B) PfeDGAT2 (D2), (C) PfeTAGLipase-like-1 (TL) , and (D) Average HFA content of all the three individual lines of each gene overexpressor (RcFAH n-value 9; D1-1 n-value 13; D2 n-value 15; TL n-value 24). Asterisks (*) and (****) indicate significant differences at p=0.0175 and P≤0.0001 compared to their respective control by one-way ANOVA.

Supplementary Table S1. List of primers used in this study

Primer name	Sequence (5'-3)	Purpose
2SProm-SeqFor2	CTTACACGTGATTGCCATGCAAATC	Squencing
F2I-5SEQR	CGAAAATGAGAAGAAATGGAAGAGAAGC	Squencing
F2I-3SEQF	CCATAGTCTTGAGTTTTCAGCTTGTTG	Squencing
GlycininTerm-SeqR2	CAGTGAACAAAAGGCAAGCTAAAGCC	Squencing
Pfe18S_F	GAGAAACGGCTACCACATCCA	qRT-PCR
Pfe18S_R	CCGTGTCAGGATTGGGTAATTT	qRT-PCR
PfeDGAT1-RNAiF	TACTACCGCGGCGGCCGCCTTCCTTTACCGTTGAGAAATTG	RNAi
PfeDGAT1-1RNAiR	GACTTCTGCAGCCCGGGGTTCGTGTGAGCATAAGAAACC	RNAi
PfeTAGLipL1RNAiF	TACTACCGCGGCGGCCGCACCTCTCGGGATGGGAATAC	RNAi
PfeTAGLipL1RNAiR	GACTTCTGCAGCCCGGGCTCGAATGTTCGATGGGAAT	RNAi
PfeDGAT2RNAiF	TACTACCGCGGCGGCCGCTGGTGTCCAGGAGACTTATCA	RNAi
PfeDGAT2RNAiR	GACTTCTGCAGCCCGGGAGATTGGGGTAAACCTGATTG	RNAi
PfeDGAT1-qPCR_F	ACAAGAAAGGTTCGGCTCAACG	qPCR
PfeDGAT1-qPCR_R	GGTTGATCCTTTGCGGTTCATC	qPCR
PfeDGAT2-qPCR_F	TTCACGTCGAGGATTACGATGC	qPCR
PfeDGAT2-qPCR_R	TCGGTAACACCGAATGTGGTTC	qPCR
PfePDAT1-qPCR_F	ATATGGGGCGGTCTTGATTG	qPCR
PfePDAT1-qPCR_R	ACTCCATTTTCGCCTGCTTC	qPCR
PfeTAGL1-qPCR_F	GGATCGATGAAGCTCAATGC	qPCR
PfeTAGL1-qPCR_R	CGGATGAAAAGCAGAGGATG	qPCR
