# Supplementary material for: Identification of triacylglycerol remodeling mechanism to synthesize unusual fatty acid containing oils
Source: Nat Commun. 2024 Apr 26;15:3547. doi: 10.1038/s41467-024-47995-x (PMC11053099; doi:10.1038/s41467-024-47995-x)
Supplement: Supplementary file 3 — Reporting Summary [file 41467_2024_47995_MOESM3_ESM.pdf]

nature portfolio

Corresponding author(s): Philip D. Bates

Last updated by author(s): Apr 5, 2024

# Reporting Summary

Nature Portfolio wishes to improve the reproducibility of the work that we publish. This form provides structure for consistency and transparency in reporting. For further information on Nature Portfolio policies, see our Editorial Policies and the Editorial Policy Checklist.

## Statistics

For all statistical analyses, confirm that the following items are present in the figure legend, table legend, main text, or Methods section.

| n/a | Confirmed | |
|---|---|---|
| ☐ | ☒ | The exact sample size (*n*) for each experimental group/condition, given as a discrete number and unit of measurement |
| ☐ | ☒ | A statement on whether measurements were taken from distinct samples or whether the same sample was measured repeatedly |
| ☐ | ☒ | The statistical test(s) used AND whether they are one- or two-sided<br>*Only common tests should be described solely by name; describe more complex techniques in the Methods section.* |
| ☒ | ☐ | A description of all covariates tested |
| ☒ | ☐ | A description of any assumptions or corrections, such as tests of normality and adjustment for multiple comparisons |
| ☐ | ☒ | A full description of the statistical parameters including central tendency (e.g. means) or other basic estimates (e.g. regression coefficient) AND variation (e.g. standard deviation) or associated estimates of uncertainty (e.g. confidence intervals) |
| ☐ | ☒ | For null hypothesis testing, the test statistic (e.g. *F*, *t*, *r*) with confidence intervals, effect sizes, degrees of freedom and *P* value noted<br>*Give P values as exact values whenever suitable.* |
| ☒ | ☐ | For Bayesian analysis, information on the choice of priors and Markov chain Monte Carlo settings |
| ☒ | ☐ | For hierarchical and complex designs, identification of the appropriate level for tests and full reporting of outcomes |
| ☒ | ☐ | Estimates of effect sizes (e.g. Cohen's *d*, Pearson's *r*), indicating how they were calculated |

*Our web collection on statistics for biologists contains articles on many of the points above.*

## Software and code

Policy information about availability of computer code

| | |
|---|---|
| Data collection | Open lab chemstation edition for GC systems version 38 (30-May-2018) C.01.09. |
| Data analysis | GraphPad Prism Version 10.1.2 was used for all statistical analysis and graphing. |

For manuscripts utilizing custom algorithms or software that are central to the research but not yet described in published literature, software must be made available to editors and reviewers. We strongly encourage code deposition in a community repository (e.g. GitHub). See the Nature Portfolio guidelines for submitting code & software for further information.

## Data

Policy information about availability of data

All manuscripts must include a data availability statement. This statement should provide the following information, where applicable:

- Accession codes, unique identifiers, or web links for publicly available datasets
- A description of any restrictions on data availability
- For clinical datasets or third party data, please ensure that the statement adheres to our policy

Data supporting the findings of this work are available within the paper and its supplementary information and source data files. A reporting summary for this article is available as a supplementary information file. The datasets and plant materials generated and analyzed during the current study are available from the corresponding author upon request. The nucleotide sequence of genes used in this study are available from the GenBank under the accession numbers: PP588719 – PfeDGAT1; PP588720 – PfeDGAT2; PP588721- PfeTAGL1.

## Research involving human participants, their data, or biological material

Policy information about studies with human participants or human data. See also policy information about sex, gender (identity/presentation), and sexual orientation and race, ethnicity and racism.

| | |
|---|---|
| Reporting on sex and gender | N/A |
| Reporting on race, ethnicity, or other socially relevant groupings | N/A |
| Population characteristics | N/A |
| Recruitment | N/A |
| Ethics oversight | N/A |

Note that full information on the approval of the study protocol must also be provided in the manuscript.

# Field-specific reporting

Please select the one below that is the best fit for your research. If you are not sure, read the appropriate sections before making your selection.

☒ Life sciences ☐ Behavioural & social sciences ☐ Ecological, evolutionary & environmental sciences

For a reference copy of the document with all sections, see nature.com/documents/nr-reporting-summary-flat.pdf

# Life sciences study design

All studies must disclose on these points even when the disclosure is negative.

| | |
|---|---|
| Sample size | Enzyme assays were done with at least 3 independent assays, all transgenic plants were screened for phenotypes through 6-20 independent transformants, and at least the top 3 lines used for experiments. All samples sizes are listed in the figure legends. Sample sizes where chosen based on standards in the filed. |
| Data exclusions | No data excluded, independent transgenic lines that have little to no phenotype are also included in the supplement. |
| Replication | All data points indicate independent enzyme assays or independent transgenic plants with at least 3 replicates. All replicates were successful. |
| Randomization | All plants including controls were grown together in the same growth chamber randomized across the growing space to account for within chamber microenvironments. |
| Blinding | No blinding |

# Reporting for specific materials, systems and methods

We require information from authors about some types of materials, experimental systems and methods used in many studies. Here, indicate whether each material, system or method listed is relevant to your study. If you are not sure if a list item applies to your research, read the appropriate section before selecting a response.

### Materials & experimental systems

| n/a | Involved in the study | |
|---|---|---|
| ☐ | ☒ | Antibodies |
| ☒ | ☐ | Eukaryotic cell lines |
| ☒ | ☐ | Palaeontology and archaeology |
| ☒ | ☐ | Animals and other organisms |
| ☒ | ☐ | Clinical data |
| ☒ | ☐ | Dual use research of concern |
| ☐ | ☒ | Plants |

### Methods

| n/a | Involved in the study | |
|---|---|---|
| ☒ | ☐ | ChIP-seq |
| ☒ | ☐ | Flow cytometry |
| ☒ | ☐ | MRI-based neuroimaging |

## Antibodies

| | |
|---|---|
| Antibodies used | mouse anti-6x-His tag monoclonal antibody (HIS.H8, Invitrogen USA; Cat.no:MA1-21315); anti-mouse IgG secondary antibody tagged |

| | |
|---|---|
| Antibodies used | with HRP (Invitrogen, USA; Cat.no: 61-6520) |
| Validation | Antibodies used are standard commercial antibodies for common epitopes and were not further validated. The specificity is demonstrated in Figure 5 as there is no western signal in the control lane. |

# Dual use research of concern

Policy information about dual use research of concern

## Hazards

Could the accidental, deliberate or reckless misuse of agents or technologies generated in the work, or the application of information presented in the manuscript, pose a threat to:

| No | Yes | |
|---|---|---|
| ☒ | ☐ | Public health |
| ☒ | ☐ | National security |
| ☒ | ☐ | Crops and/or livestock |
| ☒ | ☐ | Ecosystems |
| ☒ | ☐ | Any other significant area |

## Experiments of concern

Does the work involve any of these experiments of concern:

| No | Yes | |
|---|---|---|
| ☒ | ☐ | Demonstrate how to render a vaccine ineffective |
| ☒ | ☐ | Confer resistance to therapeutically useful antibiotics or antiviral agents |
| ☒ | ☐ | Enhance the virulence of a pathogen or render a nonpathogen virulent |
| ☒ | ☐ | Increase transmissibility of a pathogen |
| ☒ | ☐ | Alter the host range of a pathogen |
| ☒ | ☐ | Enable evasion of diagnostic/detection modalities |
| ☒ | ☐ | Enable the weaponization of a biological agent or toxin |
| ☒ | ☐ | Any other potentially harmful combination of experiments and agents |

# Plants

| | |
|---|---|
| Seed stocks | Physaria, Arabidopsis, and N. benthamina lines were from prior publications listed in the manuscript. |
| Novel plant genotypes | Arabidopsis transgenics were produced by floral dip as described in the manuscript, and 14 or more independent transformants were screened for fatty acid phenotypes and data shown in the supplement, for the main text the top 3-4 lines are displayed. Physaria transgenics were produced through tissue culture as described in the manuscript, six or more independent plant lines were screened for fatty acid phenotypes (displayed in the supplement) and the top 3 lines used for the main text. |
| Authentication | Single T-DNA homozygous lines were determined by 3:1 segregation of the DsRed selectable marker at the T2 stage. For RNAi_lines the change in expression of the gene of interest was confirmed by qPCR. |
